# The University of London

**Published:** 1903-04-25

**Authors:** 


### The University of London.

There have been indications for some time past of a growing uneasiness among the graduates of the University of London lest the Senate, in an excess of generosity, should establish certain alterations in the curricula of the examinations, the effect of which might be to lower the standard of acquirements which has hitherto been demanded from candidates presenting themselves for the different degrees, thereby lessening the worth and esteem in which these degrees have up to now been regarded by the world at large. That there were grounds for such anticipation is now demonstrated by the fact that the Senate has definitely resolved to embark upon certain radical changes which must, it would appear, have some effect upon those individuals who possess London degrees obtained under the old regime, inasmuch as the general trend of the new regulations seems to be towards a relaxation of the rigour which has been the feature of the London University examinations in the past. The alterations which are most severely criticised are (1) the decision of the Senate to confer in the future honorary degrees on individuals by selection; (2) the abolition of a separate examination, involving a test of the candidate's ability to perform operations on the dead body for the degree of bachelor of surgery; (3) the rendering it possible for those who hold lower degrees of certain foreign universities to proceed straightway to the higher degrees of the University of London. The opposition to these changes is without doubt based mainly upon an apprehension that the direct result will be to diminish to a greater or less extent the value of the London degrees and to lower the prestige of the University. The individual who at present can place the M.D.Lond. after his name, has in his possession that which for all practical purposes is a certificate of proficiency not only in medicine but in all the sciences upon which the art of medicine is based. Under the new regulations this will no longer be the case, for it will then be possible to graduate M.D.Brux., for example, and proceed subsequently to the M.D.Lond. without first undergoing any such searching examination in the elementary sciences. Moreover, there is a further point which seems likely to arise if the new regulations come into force, and that is with regard to the Royal College of Surgeons of England and Royal College of Physicians of London. How can the University of London in justice permit graduates of certain foreign universities to proceed immediately to take a London degree while refusing to grant the same privilege to the individual who holds the college diplomas and can sign M.R.C.S.Eng., L.R.C.P.Lond. after his name, seeing that the M.R.C.S.Eng., L.R.C.P.Lond. requires a higher standard of attainments and represents more value than many of the foreign degrees in medicine which the University of London has at present determined to recognise.

It is a pity that regulations which involve such drastic changes should have been passed before the matter relating to final degrees was completely settled. It is suggested that if the University of London is to widen the scope of its field that it must adopt degrees of two standards, and it is also said that such a course will effectively answer the objections which have been raised against the new regulations. It has been proposed that the Senate should establish an honours degree to be attained in the usual manner and not awarded to selected candidates, and that this honours degree should bear with it the guarantee that the recipient has passed all the tests necessary to prove the highest qualifications. The pass degree might then be associated with less exacting requirements. Such a course would probably satisfy the murmuring graduate under the old régime, as it would add to and not diminish the value of his connection with the University; it would give the ambitious and more brilliant members the opportunities they require; not bar the way to the men of average attainments. It is, however, doubtful whether it is a step in the right direction to confer a degree without insisting on a test of the candidate's general knowledge and acquirements, and it appears more than doubtful if the abolition of a practical examination in operative surgery for the B.S. can be justified. We fully sympathise with reform on the widest basis, but with one which, whilst giving in one direction, does not give up in another. London cannot afford to lower the standard of its degrees, for on this its prestige rests, and through which it has so far held its own in competition with rival Universities.

